# To the Editors of the Medical and Physical Journal

**Published:** 1801-10

**Authors:** William Blair

**Affiliations:** Great Russel Street, Bloomsbury


### *To the Editors of the Medical and Phyſical Journal.*

GENTLEMEN,

IMPERFECT and erroneous accounts having been publiſhed, reſpecting ſeveral inſtitutions eſtabliſhed in London, for the relief of the ſick-poor; a correct ſtatement would probably be deemed an acceptable preſent to the public, as well as to the medical world. It is my intention to collect and print the beſt account I am able, of all the HOSPITALS, INFIRMARIES, DISPENSARIES, and MEDICAL SOCIETIES in the Metropolis. I have therefore taken the liberty to addreſs you, in hopes of being furniſhed, through the aſſiſtance of your correſpondents, with ſuch materials as will be likely to facilitate the execution of my deſign.

The information which I particularly requeſt, may be comprehended under the following heads:

1. Original foundation of the inſtitution.
2. Subſequent alterations and improvements.
3. Preſent eſtabliſhment and income.
4. Names and writings of the medical officers.
5. Plan of medical attendance.
6. Copy of the pharmacopœia pauperum.
7. Houſe diet, nurſing, and economy.
8. Weekly board, committees, and general meetings.
9. Regulations for viſitors and attending pupils.
10. Stated Medical Lectures and Societies.
11. Publications tending to throw light on this inquiry.

I am, &c.
WILLIAM BLAIR.

